# Effect of White Tea (*Camellia sinensis*) Extract on Skin Wound Healing Process in Rats

**DOI:** 10.29252/wjps.10.1.85

**Published:** 2021-01

**Authors:** Golnoush Kouhihabibidehkordi, Soleiman Kheiri, Iraj Karimi, Fatemeh Taheri, Elham Bijad, Mohammad Bahadoram, Zahra Alibabaie, Shirin Asgharian, Hajar Zamani, Mahmoud Rafieian-Kopaei

**Affiliations:** 1 Medical Plants Research Center, Faculty of Medicine, Shahrekord University of Medical Sciences, Shahrekord, Iran;; 2 Department of Biostatistics and Epidemiology, Faculty of Health, Shahrekord University of Medical Sciences, Shahrekord, Iran;; 3 Department of Pathology, Faculty of Veterinary Medicine, Shahrekord University, Shahrekord, Iran;; 4 Department of Pathology, Faculty of Medicine, Shahrekord University of Medical Sciences, Shahrekord, Iran;; 5 Medicinal Plant Research Center, Ahvaz Jundishapur University of Medical Sciences, Ahvaz, Iran.

**Keywords:** Antioxidant, Camellia sinensis, Cutaneous wound, Healing process, White tea

## Abstract

**BACKGROUND:**

White tea (*Camellia sinensis*) has anti-inflammatory and antioxidant properties and a protective effect against wrinkles, sunburn and UV damages on the skin. Thus, we aimed to evaluate the effect of white tea extract on the healing process of skin wounds in rats.

**METHODS:**

This study was done in the Research Center of Shahrekord University of Medical Sciences, Shahrekord, Iran in 2019. Excisional skin wounds were created on five groups of healthy male Wistar rats (200-250 g, n=21) including control group, Eucerin-treated group, white tea 5% ointment (Eucerin) treated group, gel-treated group, white tea 5% gel treated group. Treatment was begun on day 1 and repeated every day at the same time until day 15. Pathologic samples were taken on days 4, 7 and 15 for histopathological examinations. Kruskal-Wallis test was used to analyze data by SPSS. Statistical significance was defined as *P*<0.05.

**RESULTS:**

Wound closure rate of control group was more than other groups on day 4 (*P*<0.05). On day 7, reepithelisation and granulation tissue of control group were more than white tea 5% ointment-treated and its inflammation was less than others (*P*<0.05). Neo-vascularization of white tea 5% ointment-treated group was more than control group on days 4 and 15 (*P*<0.05). On day 4, intact mast cells of control group were more than white tea treated groups (*P*<0.05). Degranulated mast cells of white tea 5% gel treated group was significantly (*P*<0.05) more than control group on days 4 and 15.

**CONCLUSION:**

Five percent white tea extract could not help the skin wound healing process.

## INTRODUCTION

Skin protects our bodies against dehydration and foreign pathogens influx and maintains body homeostasis and temperature ^1^^, ^^2^. Therefore, wounds by causing a disturbance in skin anatomy and function may lead to complications such as infection and even shock ^3^^, ^^4^. Because of the importance of healthy skin, wounds prevalence and the costs of skin wound treatment, it is essential to find a low-cost and effective treatment for skin wounds ^2^^,^^5^^,^^6^.

Chemical drugs are expensive and have side effects, while, medicinal plants are safe and cheap ^7^^-^^9^. Thus, the wound-healing effects of some medicinal plants have been studied and proven ^4^.

The extracts of some medicinal plants such as *Lemon, Aloe vera, Jojoba, *and *Ginseng *were used for wound healing and their common feature was the production of flavonoid compounds with phenolic structures ^10^. Flavonoids and polyphenols have anti-oxidative effect ^11^.

In a normal physiological state, production and neutralization of reactive species are in balance ^12^. Overproduction of ROS is cytotoxic and delays wound healing process. Therefore, one way to improve wound healing is reducing ROS ^5^.

The genus *Camellia* is a member of the *Theaceae* family ^13^. Tea (*C. sinensis L*) has various beneficial effects including antioxidant, anti-diabetic, neuroprotective, hypocholesterolemic, antimicrobial and antifungal activities ^14^^,^^15^. It also prevents cancers and heart disease and protects our body from Cd and Pb accumulation ^16^. *C. sinensis* water extract has anti-wrinkle effects ^17^.

Different types of tea are classified based on their formation process. White and green tea are non-fermented, red tea is semi-fermented and black tea is fermented ^14^^,^^15^^,^^18^. There are polyphenol oxidase and other oxidative enzymes in the leaves of tea; thus, during the fermentation process from white tea to black tea, the polyphenols of *C. sinensis’ *leaves become more and more oxidized ^14^^,^^18^. Therefore, white tea has the most antioxidants among all types of teas ^15^. The antioxidant property of tea seems to be due to tea flavonoids and polyphenols ^17^. Some of the features of polyphenols are antioxidant, antineoplastic and anti-inflammatory effects ^15^^, ^^19^. Moreover, they are good for skin health ^19^.

The catechins of white tea are catechin (C), epigallocatechin gallate (EGCG), epicatechin gallate (ECG), epigallocatechin (EGC) and epicatechin (EC) ^20^. They are anti-oxidants that improve wound healing by increasing collagen volume and keratinocytes reproduction ^21^. 

Considering similarities between green tea and white tea, and benefits of green tea ethanolic extract on the surgical and burn wounds ^1^^,^^21^, more anti-oxidants in white tea in comparison with green tea and the positive effects of antioxidants and antimicrobial agents on the wound healing process and all the properties of white tea, we aimed to evaluate the effect of white tea extract on the healing process of excisional skin wounds in rats ^2^^,^^14^^,^^15^^,^^18^.

## MATERIALS AND METHODS

After obtaining approval from Ethics Committee of Shahrekord University of Medical Sciences, this experiment was carried out in the Research Center of Shahrekord University of Medical Sciences in 2019.

***Preparation of white tea extract***

The Maceration method was used for preparing the extract. Dried leaves of white tea (Lahijan Refah tea, TEAMAN CO., Iran. Herbarium No. 1020, Medical Plants Research Center, SKUMS) were pulverized by a grinder machine. A mixture of 100 gr of white tea powder and 1 L of ethanol 70% (Hamoon Teb Markazi Pharmaceutical Chemical Industrial, Iran.) was made in an Erlenmeyer and kept for 48 h in the laboratory temperature. Subsequently, all the materials were filtered by filter paper, evaporated by a rotary and dried by a heater ^21^.

The powder of extract was uniformly mixed with Eucerin (Orand Eucerin, Abi Darya Co., Iran) to prepare Eucerin-based 5% ointment. A gel containing 5% extract of white tea was produced, too.

***Animals***

Healthy male Wistar rats (105 rats, 200-250 g) were housed in the same situation for light, food, and water. A standard glass cage was allocated to each rat. After acclimatization to the environment of the laboratory, 105 rats were randomly divided into five groups containing 21 rats each: control group, vehicle (Eucerin)-treated group, white tea 5% ointment (Eucerin)-treated group, vehicle (gel)-treated group and white tea 5% gel-treated group.

***Surgical wounds***

On day 0, all the rats were anesthetized with an intramuscular injection of 20 mg/kg ketamine 10% (Merck, Germany) and 2 mg/kg xylazine 2% (Merck, Germany). Then, after the disinfection of the skin of dorsal surface of their neck by betadine 10% (Behvazan pharmaceutical Co. Iran) and shaving the hair, a full-thickness circular excisional skin wound was created with 1 cm diameter on the dorsal surface of the neck by a sterile scissor ^21^^, ^^22^.

***Treatments***

All the rats were treated every day for 15 d and the treatment began on day 1 which means one day after the surgery. Every day, all the wounds of all rats were washed by normal saline serum (Daru-Pakhsh, Iran) and after that, all of them, except the control group, was given a layer of their local treatment which covered the wound completely. Therefore, the wounds of vehicle (Eucerin)-treated group were treated with the Eucerin vehicle, the wounds of white tea 5% ointment (Eucerin)-treated group with white tea 5% ointment, the wounds of vehicle (gel)-treated group with vehicle gel and wounds of white tea 5% gel treated group with gel 5% of white tea.

***Data collecting***

Seven rats from each group were randomly selected on days 4, 7 and 15. The surface of the remaining wound of each rat was drawn on a transparent paper and with the aid of AutoCAD software, the area of the remaining wound was measured. The following equation was used for wound closure rate calculation.

Wound closure rate %= [(area at day 0 – area at each day of measurement)/area at day 0] ×100 

For the histological study, the rats were euthanized and then full-thickness remaining wounds with the surrounding healthy skin were sampled and fixed in tubes containing formalin buffer 10% ^21^^, ^^22^. Subsequently, tissues were dehydrated and processed for paraffin and with the means of microtome, 5 µm thick incisions were made and then stained by Toluidine Blue, and Hematoxylin and Eosin (H&E) ^21^^, ^^23^.

The evaluation of re-epithelialization, neovascularization, granulation tissue, inflammation and collagen alignment of the samples were accomplished by a pathologist who was blinded in grouping by evaluating H&E stained tissues and the given scores were based on Table 1.

**Table 1:** System of histological scoring of samples

Histological features	Score				
**Granulation tissue**	_	Thin granular layer	Moderate granular layer	Thick granular layer	Very thick granular layer
**Re-epithelialization**	No epithelial organization	Just basal layer formed	Moderate epithelial organization	Keratinization is done	_
**Neo-vascularization**	zero formed capillary vessels	Newly formed capillary vessels<3	3-6 formed capillary vessels	formed capillary vessels>6	_
**Collagen alignment**	_	25% of collagen tissue is parallel to wound surface	25-50% of collagen tissue is parallel to wound surface	50-75% of collagen tissue is parallel to wound surface	More than 75% of collagen tissue is parallel to wound surface
**Inflammation**	No inflammation	Mild	Moderate	Sever	_

A pathologist who was blinded in grouping quantified the Mast cells of tissues with the use of Toluidine Blue stained samples.

***Data analysis***

The normal distribution of the variables was evaluated using the one-sample Kolmogorov-Smirnov test. Because the data were not distributed normally, the medians with interquartile range (IQR) were used for presenting the data. Kruskal-Wallis test followed by Dunn’s test was used to compare variables between the groups. Statistical significance was defined as *P*<0.05 and analysis was performed by SPSS version 23 (Chicago, IL, USA).

***Ethics***

The experiment was approved by the Ethical Committee of Shahrekord University of Medical Sciences (ethical code: IR.SKUMS.REC.1396.22). We followed the ethics principles of working on animals.

## RESULTS

***Measurement of phenolic and flavonoid compounds***

The phenolic compounds were totally 226.45 mg/g in Gallic acid equivalent.

The flavonoid compounds were totally 9.95 mg Rutin equivalent/g.

***Wound closure rate***

Median with Interquartile ranges (IQR) of wound closure rates in groups are shown in Table 2. 

**Table 2:** Median with Interquartile range (IQR) of wound closure rates in groups

Group	Day4	Day7	Day15
**Control**	69.5(66.6-73.6)*	61.6(58-86.4)	100(97.8-100)
**Eucerin-treated**	51.1(48.1-61.8)*	77.3(75-81.9)	100(99-100)
**White tea 5% ointment-treated**	46.1(41.4_58.9)*	65(55.4-70.2)	100(100-100)
**Gel-treated**	60.9(48.4-65)	76(67.3-83.7)	99.5(98-100)
**White tea 5% gel-treated**	50.8(36.8-57)*	82(81-85.8)	100(100-100)

On day 4, the wound closure rate of control group was significantly (*P*<0.05) more than Eucerin-treated group, white tea 5% ointment (Eucerin)-treated group and white tea 5% gel treated group.

***Histological findings***

**Fig. 1:**
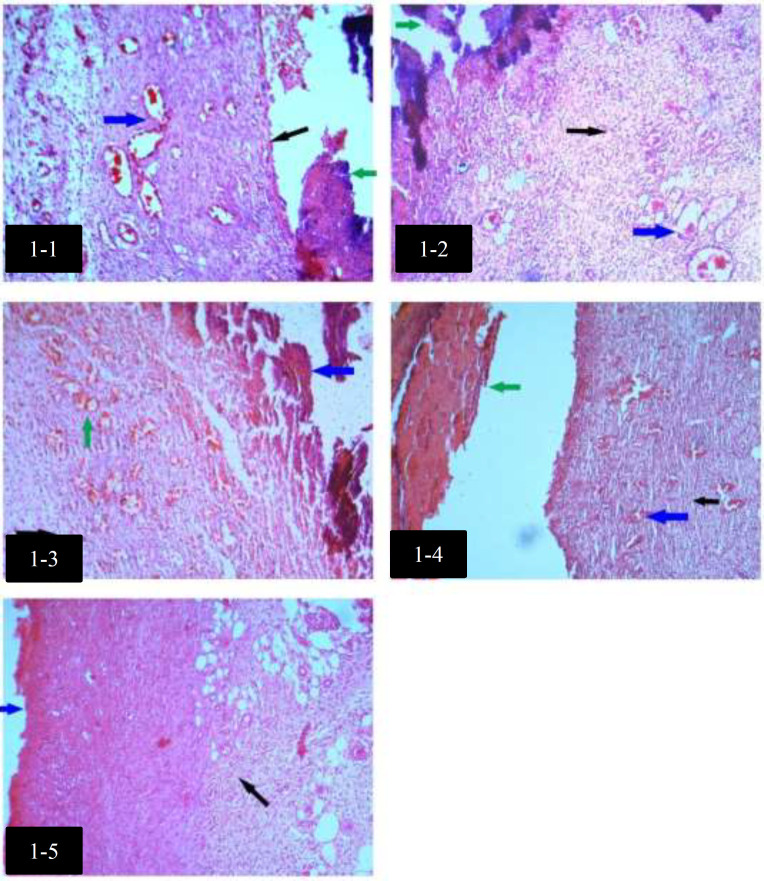
Histological differences among groups with H&E staining, on day 4

**Fig. 2:**
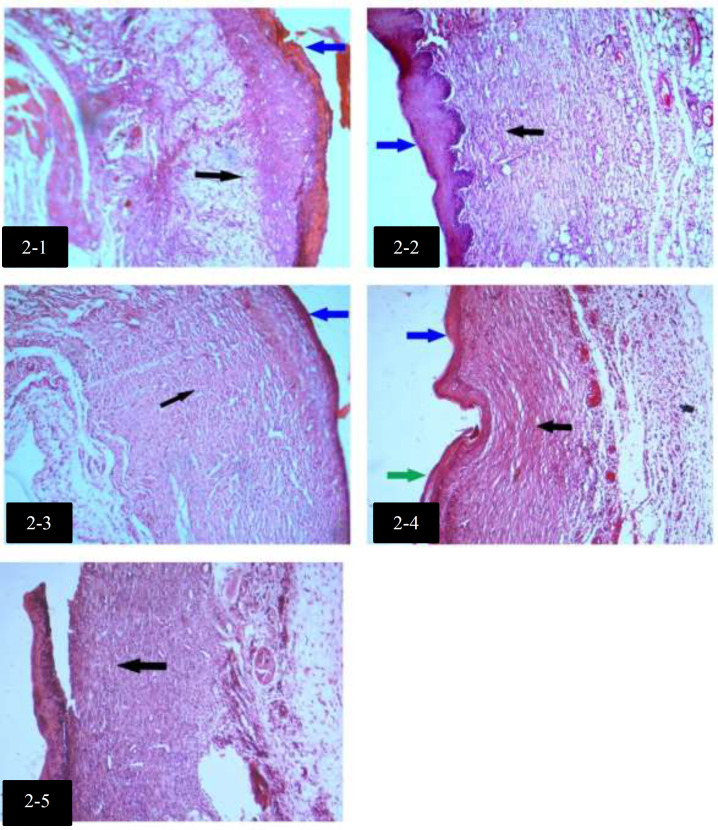
Histological differences among groups with H&E staining, on day 7

Median with Interquartile ranges (IQR) of histological findings in groups are shown in Table 3. There was no significant difference in Collagen alignments between groups. Histological differences among groups with H&E staining, on day 4, are shown in Figure 1. Histological differences among groups with H&E staining, on day 7, are shown in Figure 2.

**Table 3:** Median with Interquartile range (IQR) of histological findings in groups

Group		Granulation tissue	Re-epithelialization	Neo-vascularization	Collagen alignment	Inflammation
**Control**	Day 4 Day 7 Day 15	2(2-2) 3(3-4)❶ 3(3-3)❷	0(0-0) 1(1-2)❸ 3(2/5-3)	2(2-2)❺ 2(2-3) 0(0-1)❻	1(1-1) 2(2-3) 3(2/5-3)	2(1-2) 0(0_0)❼ 0(0_0)
**Eucerin-treated**	Day 4 Day 7 Day 15	2(1-2) 3(2-3) 3(2-3)	0(0-2) 0(0-2) 3(2-3)	2(2-2) 2(2-3) 1(1-2)	1(1-1) 2(1-2) 2(1-3)	1(1-1) 1(0-1)❼ 0(0_0)
**White tea 5% ointment-treated**	Day 4 Day 7 Day 15	2(2-2) 2(2-3)❶ 3(3-3)	0(0-0) 0(0-0/25)❸ 3(3-3)	3(2-3)❺ 3(2-3) 1(1-2)❻	1(1-1) 2(1-2) 3(3-3)	2(1-2) 1(1-1)❼ 0(0_0)
**Gel-treated**	Day 4 Day 7 Day 15	2(1/5-3) 3(3-4) 4(3/75-4)❷	0(0-0) 3(2-3)❹ 3(2/25-3)	2(1/5-3) 2(1/5-2/5) 1(0/75-2)	1(0/5-2) 3(2/5-3) 4(2/75-4)	1(0/5-1/5) 0(0-1) 0(0_0/25)
**White tea 5% gel-treated**	Day 4 Day 7 Day 15	2(2-2) 3(3-4) *	0(0-0) 0(0-0)❹ 3(3-3)	1(1-2) 2(1-3) *	1(1-2) 2(2-2) *	2(1-2) 1(0-2)❼ 0(0_0)

*: IQR was not calculable due to the lack of enough data.

On day 7, the granulation tissue of control group was significantly (P<0.05) more than white tea 5% ointment (Eucerin)-treated group (❶).

On day 15, the granulation tissue of vehicle (gel)-treated group was significantly (P<0.05) more than control group (❷).

On day 7, the re-epithelialization of control group was significantly (P<0.05) more than white tea 5% ointment (Eucerin)-treated group (❸).

On day 7, the re-epithelialization of vehicle (gel)-treated-group was significantly (P<0.05) more than white tea 5% gel-treated group (❹).

On days 4 and 15, the neovascularization of white tea 5% ointment (Eucerin)-treated group was significantly (P<0.05) more than control group (❺, ❻).

On day 7, the inflammation of control group was significantly (P<0.05) less than vehicle (Eucerin)-treated group, white tea 5% ointment (Eucerin)-treated group and white tea 5% gel-treated group (❼).

1-1: Granulation tissue formation with abundant blood vessels (blue arrow), no epidermis (black arrow) and presence of superficial clot (green arrow) in control group on day 4 (H&E, X10).

1-2: Granulation tissue formation with irregular collagen fibers (black arrow), abundant blood vessels (blue arrow), no epidermis and presence of superficial clot (green arrow) in Eucerin-treated group on day 4 (H&E, X10).

1-3: Filling of the wound with granulation tissue and irregular collagen fibers (black arrow), abundant blood vessels (green arrow), no epidermis and presence of superficial clot (blue arrow) in white tea 5% ointment (Eucerin)-treated group on day 4 (H&E, X10).

1-4: Filling of the wound with granulation tissue and regular collagen fibers (black arrow), abundant blood vessels (blue arrow), no epidermis and presence of superficial clot (green arrow) in gel-treated group on day 4 (H&E, X10).

1-5: Filling of the wound with granulation tissue and partially regular collagen fibers (black arrow), no superficial epidermis (blue arrow) in white tea 5% gel-treated group on day 4 (H&E, X10).

2-1: Filling of the wound with granulation tissue and irregular collagen fibers (black arrow), no epidermis and presence of superficial clot (blue arrow) in control group on day 7 (H&E, X10).

2-2: Filling of the wound with granulation tissue and regular collagen fibers (black arrow), epidermis formation (blue arrow) in Eucerin-treated group on day 7 (H&E, X10).

2-3: Filling of the wound with granulation tissue and regular collagen fibers (black arrow), epidermis formation (blue arrow) in white tea 5% ointment (Eucerin)-treated group on day 7 (H&E, X10).

2-4: Filling of the wound with granulation tissue and regular collagen fibers (black arrow), Keratinized (green arrow) superficial epidermis formation (blue arrow) in gel-treated group on day 7 (H&E, X10).

2-5: Filling of the wound with granulation tissue and dense regular collagen fibers (black arrow) in white tea 5% gel-treated group on day 7 (H&E, X10).

**Fig. 3:**
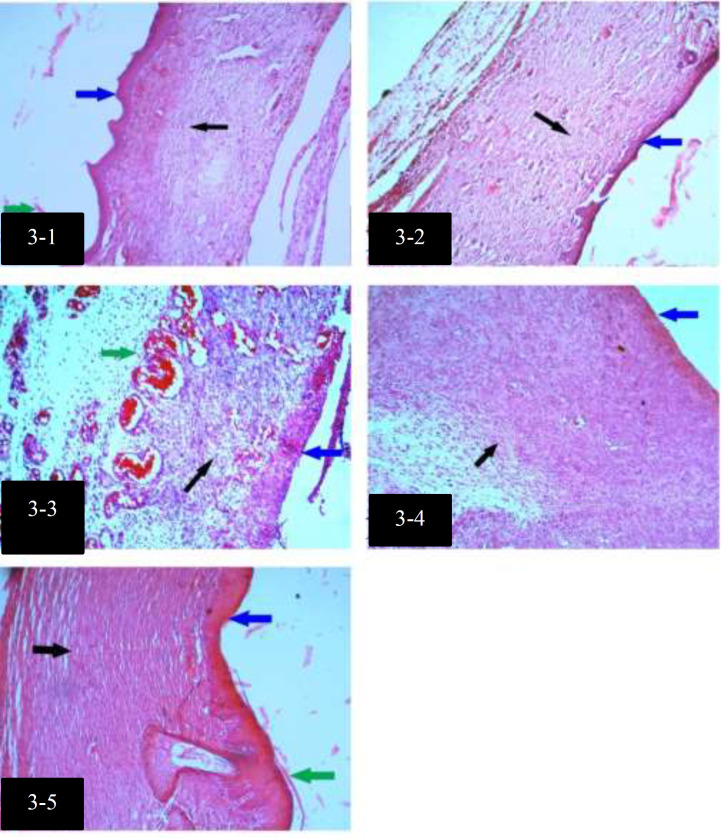
Histological differences among groups with H&E staining, on day 15

Histological differences among groups with H&E staining, on day 15, are shown in Figure 3. Median with Interquartile ranges (IQR) of intact and degranulated Mast cells in groups are shown in Table 4.

3-1: Filling of the wound with granulation tissue and regular collagen fibers (black arrow), epidermis formation (blue arrow) along with superficial keratinization (green arrow) in control group on day 15 (H&E, X10).

3-2: Filling of the wound with granulation tissue and regular collagen fibers (black arrow), superficial epidermis formation (blue arrow) in Eucerin-treated group on day 15 (H&E, X10).

3-3: Filling of the wound with granulation tissue and sparse irregular collagen fibers (black arrow), abundant blood vessels (green arrow), no superficial epidermis formation (blue arrow) in white tea 5% ointment (Eucerin)-treated group on day 15 (H&E, X10).

3-4: Filling of the wound with granulation tissue and partially regular collagen fibers (black arrow), superficial epidermis formation (blue arrow) in gel-treated group on day 15 (H&E, X10).

3-5: Filling of the wound with granulation tissue and dense regular collagen fibers (black arrow), Keratinized (green arrow) superficial epidermis formation (blue arrow) in white tea 5% gel-treated group on day 15 (H&E, X10).

**Table 4:** Median with Interquartile range (IQR) of intact and degranulated Mast cells in groups

Group		Intact Mast cells	Degranulated Mast cells
**Control **	Day 4 Day 7 Day 15	14(7-18)① 3(2-4) 3(1-3)	4(0-6)③ 4(2-6) 0(0-1)④
**Eucerin-treated**	Day 4 Day 7 Day 15	6(5-12) 2(1-3) 2(2-3)	8(2-9) 3(2-6) 1(0-2)
**White tea 5% ointment-treated**	Day 4 Day 7 Day 15	3(2-4.5)① 2(1.5-3.5) 2(2-3)	8(5-9.5) 5(3-6) 0(0-1)
**Gel-treated**	Day 4 Day 7 Day 15	4(4-4) 4(4-4) 2(1-2)②	6(3-7) * 0(0-1)④
**White tea 5% gel-treated**	Day 4 Day 7 Day 15	2(2-3)① 3(3-7) 3.5(3-4.75)②	9(7-16)③ 5(5-9) 2.5(1.25-3)④

On day 4, intact Mast cells of control group were significantly (p<0.05) more than intact Mast cells of white tea 5% ointment-treated group and white tea 5% gel treated group (①).

On day 15, intact Mast cells of white tea 5% gel treated group were significantly (p<0.05) more than intact Mast cells of gel-treated group (②).

On day 4, degranulated Mast cells of white tea 5% gel treated group were significantly (p<0.05) more than degranulated Mast cells of control group (③).

On day 15, degranulated Mast cells of white tea 5% gel treated group were significantly (p<0.05) more than degranulated Mast cells of control group and gel-treated group (④).

*: IQR was not calculable due to the lack of enough data.

**Fig. 4:**
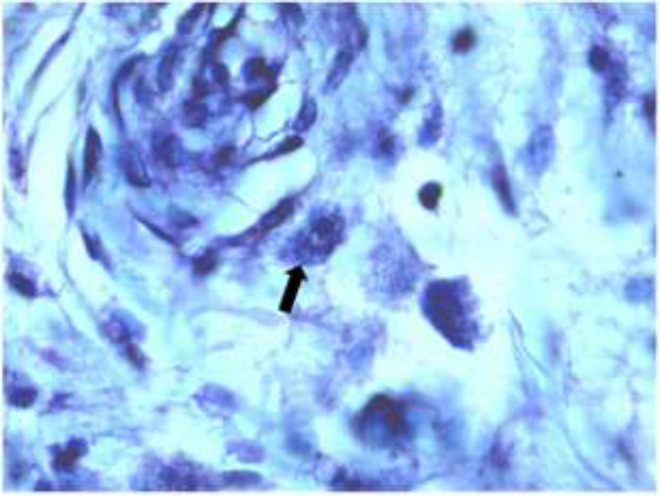
Mast cell in the wound (black arrow) (X100, Toluidine Blue)

Mast cell in the wound with Toluidine Blue staining is shown in Figure 4. 

## DISCUSSION

This study aimed to evaluate the effect of white tea extract on the wound healing process in male Wistar rats (105 rats, 200-250 g) showed that despite flavonoids and antioxidant properties of white tea, it did not improve wound healing. 

After occurrence of a wound (which is an interruption of the continuity of cells of the skin), the wound healing process begins; in which the four overlapping phases including hemostasis, inflammation, cellular proliferation, and remodeling should be done in proper sequence and time frame ^8^^, ^^24^^, ^^25^. Some factors have effects on the wound healing process; such as age, sex, stress, alcohol, smoking, nutritional condition, and diabetes ^4^.

Plant-based medicines are used by 80% of world’s population ^26^. Various plants have been studied to evaluate their effects on the wound healing process. A blend of *Alchemilla vulgaris* and *Mimosa* had positive effects on re-epithelialization, collagen synthesis and angiogenesis ^27^. Ethyl acetate extract of *Gmelina arborea *has anti-inflammatory effects and could promote keratinocytes’ wound healing ^28^. *Green tea* extract could help wound healing of the surgical wound of rats ^21^. *Iris forentina* caused higher wound closure and density of fibroblast and collagen bundles in rats ^22^. The positive effects of these plants have been attributed to their phenolic compounds, especially the flavonoid components.

Different studies showed the burn wound healing activity of polyphenols and antioxidants ^29^^-^^31^. Besides, polyphenols and white tea have a protective effect against wrinkles, sunburn and UV damages on the skin^17^^, ^^19^.

Therefore, the lack of positive effects in the present study is somewhat unexpected.

In another study, the effect of *henna, pomegranate, myrrh*, the mixture of these three and Gentamycin on wound healing was compared. The most wound contracture and the least epithelialization duration were reported in the group treated with the mixture of plants ^24^. 

Some other plants used for cutaneous wound treatment have flavonoid compounds ^10^. Several properties have been attributed to flavonoids, such as anti-inflammatory properties ^19^. Inflammation is part of normal wound healing. Neutrophils, macrophages and T lymphocytes are the main cells of this phase in which neutrophils arrive at the wound location in 24-36 h, while, macrophages and T lymphocytes in 72 hours ^32^.

Results of inflammation among the groups of this study (Table 3) showed that on day 7, inflammation in control group was significantly (*P*<0.05) less than Eucerin-treated, white tea 5% ointment (Eucerin)-treated and white tea 5% gel-treated groups; while, we do not expect inflammation until this day ^32^. Impaired wound healing has some complication such as displeasing scar formation and infection ^21^.

There are a lot of factors that can increase the risk of impaired wound healing and chronic wounds including genetic disorders like down-syndrome and ataxia-telangiectasia, aging, psychological stress by harming the immune system, malnutrition including zinc, vitamin B12, vitamin C and vitamin D deficiencies, the excess activity of macrophages through the release of pro-inflammatory cytokines, infection, diabetes through various means such as hyperglycemia, hypoxia and impaired immunity, coagulation defects, inadequate angiogenesis in patients with DM, and venous stasis and elderly ^6^^, ^^25^^, ^^32^^-^^38^

Other influential factors are foreign bodies, some diseases such as jaundice and uremia, obesity, and medications like glucocorticoids ^25^.

## CONCLUSION

White tea 5% extract did not improve the wound healing process except neovascularization, attributed to the observed inflammation. The only factor ameliorated by white tea extract was neovascularization which may be due to polyphenols and antioxidant effects of white tea. There might be additional influential factors for impaired wound healing; therefore, additional studies are needed.
